# Phospholipase D promotes *Arcanobacterium haemolyticum *adhesion via lipid raft remodeling and host cell death following bacterial invasion

**DOI:** 10.1186/1471-2180-10-270

**Published:** 2010-10-25

**Authors:** Erynn A Lucas, Stephen J Billington, Petteri Carlson, David J McGee, B Helen Jost

**Affiliations:** 1 Department of Veterinary Science and Microbiology, The University of Arizona, 1117 E Lowell Street, Tucson, AZ 85721, USA; 2 Department of Bacteriology and Immunology, Haartman Institute, University of Helsinki, Haartmaninkatu 3, P.O. Box 21, Helsinki, FIN-00014, Finland; 3 Department of Microbiology and Immunology, Louisiana State University Health Sciences Center-Shreveport, 1501 Kings Highway, Shreveport, LA 71130, USA; 4 Modesto Junior College, 435 College Avenue, Modesto, CA 95350, USA; 5 Ventana Medical Systems, Inc., 1910 Innovation Park Drive, Oro Valley, AZ 85755, USA; 6 Office for the Responsible Conduct of Research, The University of Arizona, P.O. Box 245092, Tucson, AZ 85721, USA

## Abstract

**Background:**

*Arcanobacterium haemolyticum *is an emerging bacterial pathogen, causing pharyngitis and more invasive infections. This organism expresses an unusual phospholipase D (PLD), which we propose promotes bacterial pathogenesis through its action on host cell membranes. The *pld *gene is found on a genomic region of reduced %G + C, suggesting recent horizontal acquisition.

**Results:**

Recombinant PLD rearranged HeLa cell lipid rafts in a dose-dependent manner and this was inhibited by cholesterol sequestration. PLD also promoted host cell adhesion, as a *pld *mutant had a 60.3% reduction in its ability to adhere to HeLa cells as compared to the wild type. Conversely, the *pld *mutant appeared to invade HeLa cells approximately two-fold more efficiently as the wild type. This finding was attributable to a significant loss of host cell viability following secretion of PLD from intracellular bacteria. As determined by viability assay, only 15.6% and 82.3% of HeLa cells remained viable following invasion by the wild type or *pld *mutant, respectively, as compared to untreated HeLa cells. Transmission electron microscopy of HeLa cells inoculated with *A. haemolyticum *strains revealed that the *pld *mutant was contained within intracellular vacuoles, as compared to the wild type, which escaped the vacuole. Wild type-infected HeLa cells also displayed the hallmarks of necrosis. Similarly inoculated HeLa cells displayed no signs of apoptosis, as measured by induction of caspase 3/7, 8 or 9 activities.

**Conclusions:**

These data indicate that PLD enhances bacterial adhesion and promotes host cell necrosis following invasion, and therefore, may be important in the disease pathogenesis of *A. haemolyticum *infections.

## Background

*Arcanobacterium haemolyticum *is a gram positive, non-motile rod originally identified as a cause of pharyngitis and wound infections in U.S. servicemen and Pacific islanders [1,2]. *A. haemolyticum *is almost exclusively a human pathogen, making it somewhat unique within the genus [3]. The other species are uncommonly isolated, with the exception of *Arcanobacterium pyogenes*, which is an economically important opportunistic pathogen of livestock [3].

*A. haemolyticum *pharyngitis is a disease of adolescents and young adults, with >90% of cases occurring in patients between 10-30 years of age [4-6]. Clinically, *A. haemolyticum *pharyngitis resembles that caused by *Streptococcus pyogenes*, although in 33-66% of cases, an erythematous rash occurs after onset [5,7]. More rarely, *A. haemolyticum *is responsible for invasive diseases such as meningitis [8], septic arthritis [9], and osteomyelitis [10]. Invasive infections occur in older patients (>30 years) who may be immunocompromised or have other co-morbid factors [11,12]. However, invasive infections also occur in younger, immunocompetent patients (15-30 years), who often have a prior history of upper respiratory tract disease (pharyngitis, sinusitis) due to *A. haemolyticum *[12,13]. This suggests that invasion of the organism to distal sites may occur from the initial site of infection in the nasopharynx.

Little is known about *A. haemolyticum *virulence factors and consequently, the mechanisms of pharyngeal infection and dissemination into deeper tissues remain to be elucidated. Initial virulence studies were performed by intradermal injection of bacteria into humans, guinea pigs and rabbits, resulting in elevated abscesses with necrosis and a pronounced neutrophil infiltration 24-48 hours post infection [2]. However, attempts to induce pharyngitis by inoculation of bacteria onto the human pharynx were unsuccessful [2]. Intravenous inoculation of *A. haemolyticum *into rabbits resulted in hemorrhagic pneumonia [2], suggesting this organism can cause invasive disease once it enters the bloodstream. Subsequently, a phospholipase D (PLD) was identified and shown to cause the dermonecrosis observed [14]. While the role of *A. haemolyticum *PLD in pathogenesis is currently unclear, PLD is expressed during infection, as determined by the presence of serum antibodies in pharyngitis patients [15,16].

PLDs are ubiquitous enzymes which cleave phospholipids, including phosphatidylcholine (PC) and sphingomyelin (SM), both of which are abundant in the mammalian plasma membrane [17]. SM, with cholesterol and GPI-anchored proteins, predominantly partitions to lipid rafts, which are tightly packed, membrane micro-domains that act to compartmentalize cellular processes on the outer leaflet of the plasma membrane [18]. Lipid rafts are also implicated in host cell invasion by microorganisms [19]. Host PLD cleaves SM releasing ceramide and accumulation of ceramide within rafts alters their biophysical properties, leading to the formation of large, ceramide-rich membrane platforms [20]. These platforms allow reorganization and aggregation of protein receptors and receptor-associated signaling molecules, which in turn facilitates efficient signal transduction for normal physiological processes [20]. In contrast, PC found in the liquid disordered, or non-raft, phase, is associated with both the inner and outer membrane leaflets, and is cleaved by PLD to phosphatidic acid and choline, which also have roles as second messengers [18].

PLD is the only *A. haemolyticum *virulence factor cloned and sequenced to date [21]. Almost invariantly, PLDs possess two His-X-Lys-X_4_-Asp (HKD) motifs that are involved in catalysis [22]. However, the PLD expressed by *A. haemolyticum *is not related to these more common HKD PLDs and has a limited substrate specificity which includes SM, but not PC [23], leading to the alternate nomenclature, sphingomyelinase D. Unlike host sphingomyelinases, *A. haemolyticum *PLD cleaves SM releasing ceramide-1-PO_4 _instead of ceramide. Like ceramide, ceramide-1-PO_4 _is a bioactive sphingolipid, and it acts as a signaling molecule involved in regulating critical cell functions [24].

*A. haemolyticum *PLD is most closely related to the PLD of *Corynebacterium pseudotuberculosis *[21]. In *C. pseudotuberculosis*, PLD is absolutely required for virulence, as a *pld *mutant could not spread from the site of inoculation or persist in the lymph nodes [25]. *C. pseudotuberculosis *PLD hydrolyzes SM in host cell membranes and lysophosphatidylcholine in plasma [23], which causes endothelial membrane leakage and cytolysis, leading to enhanced vascular permeability [25]. *C. pseudotuberculosis *PLD also activates complement [26], promotes neutrophil chemotaxis [27] and is directly dermonecrotic when injected into the skin [26]. The PLDs of recluse spider (*Loxosceles *spp.) venom are also structurally and functionally related to the *A. haemolyticum *and corynebacterial PLDs [28]. Purified spider PLD induces intravascular hemolysis and cytokine upregulation [29], dermonecrosis [30], and complement-mediated lysis of erythrocytes [26].

PLD expression is uncommon among other bacterial pathogens and these PLDs are exclusively of the HKD superfamily. However, most of the pathogens that do express PLD have obligate or facultative intracellular lifestyles and expression of this enzyme is thought to be involved in disease pathogenesis [31-35]. Specifically in *Neisseria gonorrhoeae *and *Rickettsia *spp., PLDs are required for invasion of host cells [32,35].

This work characterizes the effects of *A. haemolyticum *PLD on host cells, with an aim to elucidating the role of this toxic enzyme in disease pathogenesis. We report that PLD is required for optimal adhesion to host cells, via remodeling of lipid rafts. Furthermore, PLD expressed inside host cells is directly toxic, leading to cell death via necrosis. These findings provide the first conclusive evidence that PLD may be required for *A. haemolyticum *disease pathogenesis.

## Results

### Analysis of the *pld *gene region

**Figure 1:**
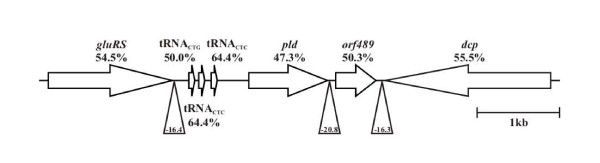
**Map of the *pld *gene region**. The open arrows indicate genes and the direction of transcription. Triangles below the sequence indicate the location of stem-loop structures, with the ΔG (kcal/mol) shown inside the triangle. Gene names are given above or below the arrows and the number below the name indicates the %G + C of the gene. A bar indicating 1-kb is shown on the right.

A draft genome sequence of *A. haemolyticum *ATCC9345 was determined (B.H. Jost and S.J. Billington, unpublished data), and this data was used to identify sequences flanking the *pld *gene (GenBank Accession Number L16583). The *pld *gene was found in a region resembling a 1.9-kb genomic island of lower %G + C than the rest of the *A. haemolyticum *genome (53.1%). This region consists of *pld *(47.2% G + C), and *orf489 *(50.3% G + C) which lacks a signal sequence and is of unknown function (Figure 1). 43-bp downstream of *pld *and 17-bp upstream of *orf489 *is a stem-loop structure with a ΔG = -20.8 kcal/mol, which may act as a transcriptional terminator or attenuator. There does not appear to be any direct or indirect repeats flanking this region. The *pld *region is flanked upstream by three tRNA genes and *gluRS*, encoding a glutamyl-tRNA synthetase (EC 6.1.1.17), and downstream by *dcp*, encoding a peptidyl-dipeptidase (EC 3.4.15.5), which is divergently transcribed compared to *pld *(Figure 1). The %G + C of the surrounding housekeeping genes (Figure 1) more closely resembles the %G + C of the *A. haemolyticum *genome.

Given the variation in %G + C of the *pld *gene and the presence of adjacent tRNA genes, which often act as sites of foreign gene insertion [36], it is possible that the *A. haemolyticum pld *gene was acquired by horizontal gene transfer. It would appear that *orf489 *is also part of the transferred DNA, and while it is not translationally coupled to *pld*, its transcription may be linked to that of *pld *despite the presence of a transcriptional terminator/attenuator between the two genes. While its function cannot be ascertained from database comparisons, it is not required for PLD activity, as this enzyme is functional when expressed from a recombinant plasmid lacking *orf489*, such as the one used as a complementing plasmid (see below).

### PLD is expressed by all isolates of *A. haemolyticum*

The prevalence of the *pld *gene was determined by DNA hybridization using a *pld*-specific gene probe. The *pld *probe hybridized at high stringency to each of 52 *A. haemolyticum *isolates, but not to *A. pyogenes *BBR1 (data not shown), indicating that *pld *is present in all strains. Furthermore, all 52 isolates express PLD as determined by a PLD activity assay (data not shown). Expression of PLD throughout the growth curve was also determined. PLD expression commenced as the bacteria entered log-phase and maximal expression was observed throughout logarithmic growth (data not shown).

### PLD stimulates lipid raft remodeling

**Figure 2:**
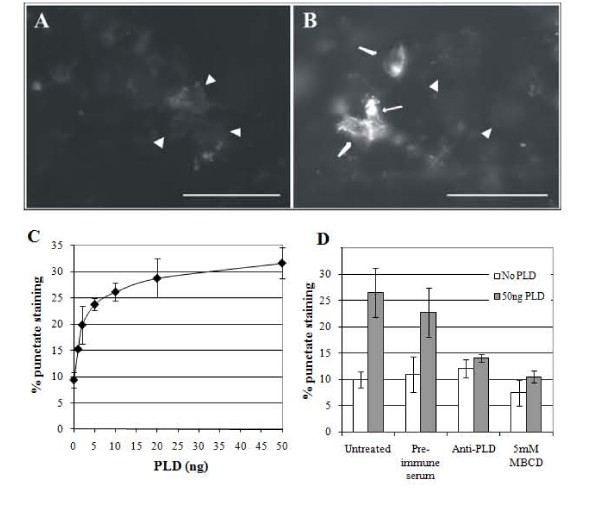
**PLD stimulates the formation of lipid rafts in a dose-dependent manner**. HeLa cells were treated (A) without or (B) with 50 ng PLD for 10 min at 37°C followed by staining with the Vybrant Lipid Raft Labeling Kit. The arrows indicate cells with bright, punctate staining, while the arrow heads indicate diffusely staining cells. Bar 50 μm. (C) HIS-PLD was added to HeLa cells for 10 min at 37°C prior to measurement of lipid raft formation. At least 100 cells were counted and the percentage of cells displaying punctate staining were enumerated. (D) Anti-PLD antibodies or the cholesterol sequestering agent MβCD inhibit PLD-mediated lipid raft formation. HeLa cells were untreated, or treated with 1/1000 dilutions of pre-immune or anti-PLD serum or 5 mM MβCD prior to addition of 50 ng HIS-PLD and measurement of lipid raft formation. Untreated HeLa cells or those treated with pre-immune, anti-PLD serum or MβCD, but not HIS-PLD served as the negative controls. Error bars indicate one standard deviation from the mean calculated from the averages of at least three independent experiments conducted in triplicate. Statistical significance was calculated using single factor ANOVA and *p *< 0.05 was considered significant.

As PLD acts on SM which is abundant in host cell lipid rafts, we hypothesized that PLD may perturb these structures, which in turn, could exacerbate the *A. haemolyticum *disease process. HeLa cells were treated with purified HIS-PLD and the ability of this toxin to cause lipid raft rearrangement was assessed. Cells displaying punctate staining were considered positive for lipid raft rearrangement (Figure 2B), whereas cells displaying a diffuse staining pattern were considered negative (Figure 2A). 9.4% of untreated, control HeLa cells displayed punctate staining (Figure 2C). Similarly, HeLa cells treated with HIS-protein purification buffer displayed similar levels of punctate staining as the control (data not shown). Upon addition of increasing amounts of HIS-PLD (0-50 ng), the number of cells with punctate staining significantly increased in a dose-dependent manner from 9.4% to 31.7% (Figure 2C).

The ability of anti-PLD antibodies to block PLD-mediated lipid raft rearrangement was investigated. In the absence of PLD, addition of pre-immune or anti-PLD serum did not significantly affect the number of punctate-staining cells compared to untreated HeLa cells (9.9%; negative control; Figure 2D). In the presence of PLD, 26.0% of HeLa cells displayed punctate staining (positive control; *p *< 0.05 compared to the negative control; Figure 2D). In the presence of PLD, addition of pre-immune serum did not significantly affect the number of punctate staining cells as compared to the positive control (*p *= 0.25; Figure 2D). In contrast, in the presence of PLD, the addition of anti-PLD antibodies significantly reduced the number of punctate staining cells (*p *< 0.05 compared to the positive control; Figure 2D). Numbers of punctate staining cells under these conditions were not significantly different to untreated HeLa cells (p = 0.15; Figure 2D), indicating that the lipid raft rearrangement seen is specific to the action of PLD.

Cholesterol sequestration by MβCD blocks lipid raft rearrangement by partially solubilizing GPI-anchored and transmembrane proteins [37] and preventing small lipid rafts from aggregating into larger, functional membrane platforms [20]. In the absence of PLD, only 9.9% of HeLa cells displayed punctate staining (untreated negative control; Figure 2D). Treatment of HeLa cells with 5 mM MβCD significantly reduced the amount of punctate staining cells to 7.4% (*p *< 0.05 compared with the negative control; Figure 2D), indicating that spontaneous lipid raft rearrangement was being inhibited. In the presence of PLD, 26.0% of HeLa cells displayed punctate staining (positive control; *p *< 0.05 compared to negative control; Figure 2D). Treatment of HeLa cells with MβCD significantly reduced the level of punctate staining to 10.5% (*p *< 0.05 compared with the positive control; Figure 2D). This value is similar to the amount of lipid raft rearrangement seen in negative control HeLa cells (9.9%; *p *= 0.54; Figure 2D). These data indicate that PLD-mediated lipid raft rearrangement can be inhibited by cholesterol sequestration.

### *A. haemolyticum *PLD is required for efficient bacterial adhesion to the host cell

The ability of PLD to enhance lipid raft rearrangement may affect the interaction between the bacterium and the host cell. To test this hypothesis, wild type and *pld *mutant *A. haemolyticum *were assayed for their ability to adhere to HeLa cells. A *pld *mutant was constructed by allelic exchange and this mutant lost its hemolytic phenotype on TS agar containing 5% bovine blood and 10% Equi Factor. Hemolysis was restored to wild type levels upon complementation with pBJ61, carrying the *pld *gene (data not shown). The hemolytic phenotype was not affected by the introduction of the shuttle vector alone (data not shown).

**Figure 3:**
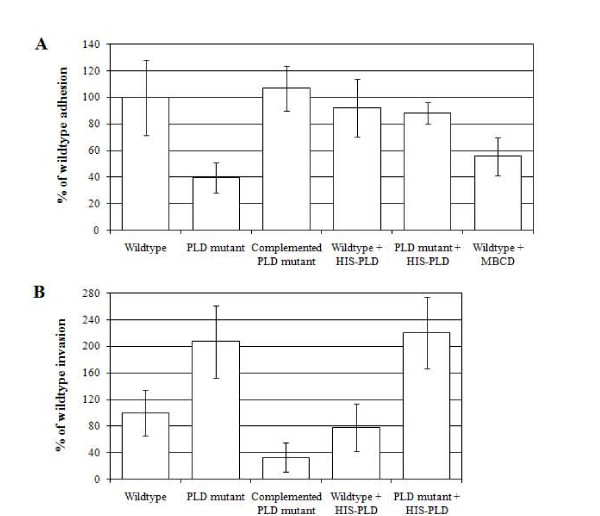
**PLD expression differentially affects adhesion (A) and invasion (B) of *A. haemolyticum *into HeLa cells**. (A) *A. haemolyticum *strains were added to cell monolayers, with or without 5 mM MβCD or 312 ng HIS-PLD, and allowed to adhere for 2 h at 37°C prior to washing and recovery of cell-associated bacteria. (B) Following adhesion, cell monolayers were washed and incubated for an additional 2 h in the presence of 10 μg/ml gentamicin to kill external bacteria. Adhesion or invasion are shown as a percentage of wild type, which was set to 100%. Error bars indicate one standard deviation from the mean calculated from the averages of at least three independent experiments conducted in triplicate. Statistical significance was calculated using single factor ANOVA and *p *< 0.05 was considered significant.

The ability of wild type or the *pld *mutants to adhere to HeLa cells was determined. Wild type *A. haemolyticum *adhered to HeLa cells at an average of 13.4% of the inoculum. This value was set at 100% and the adhesion of the other strains was determined as a percentage of wild type adhesion. The *pld *mutant was significantly impaired in adhesion, adhering at only 39.7% of the wild type (*p *< 0.05; Figure 3A). Complementation of the *pld *mutant with *pld in trans *restored adhesion to 106.9% of wild type (Figure 3A). It should be noted that the assay as performed measures both adhered bacteria and any that have subsequently invaded. However given that invasion follows bacterial adhesion, all cell-associated bacteria, whether internalized or on the cell surface, were at one point adherent to the host cell.

We hypothesized that *A. haemolyticum *PLD promoted bacterial adhesion to host cells via receptor clustering as a result of SM cleavage, leading to lipid raft signaling. Treatment of cells with 5 mM MβCD resulted in a 44.4% reduction in the adherence of wild type *A. haemolyticum *to HeLa cells, as compared to untreated controls (*p *< 0.05; Figure 3A), indicating that the loss of lipid raft rearrangement directly affected the ability of *A. haemolyticum *to adhere to HeLa cells.

### *A. haemolyticum *lacking PLD appear to invade HeLa cells more efficiently

The ability of wild type and *pld *mutants to invade host cells was also determined. Wild type *A. haemolyticum *invaded HeLa cells at an average of 0.24% of the adherent bacteria. This value was set at 100% and the invasion of the other strains was determined as a percentage of wild type invasion. The *pld *mutant was not impaired in invasion, and could invade significantly better at 207.1% of wild type *A. haemolyticum *(*p *< 0.05; Figure 3B). Complementation of the *pld *mutant led to significantly more impaired invasion than the wild type (only 33.0% of wild type; *p *< 0.05; Figure 3B), which probably results from a gene dosage effect of *pld *expressed from a multi-copy plasmid.

We also examined the effect of exogenously-added recombinant HIS-PLD on bacterial adhesion and invasion. HIS-PLD significantly enhanced the adhesion of the *pld *mutant and returned it to wild type levels (*p *< 0.05 compared to the *pld *mutant without exogenous PLD; Figure 3A). Addition of exogenous PLD did not enhance adhesion of the wild type (Figure 3A), suggesting that under these conditions, the effect of PLD on wild type adhesion is at saturation. As the exogenously-added PLD is soluble and not bacterially-associated, this indicates that PLD cannot directly act as an adhesin. Bacterial invasion was not altered in the presence of exogenous PLD for either the wild type or *pld *mutant, suggesting that PLD does not play a direct role in invasion once the bacteria are adherent (Figure 3B).

### HeLa cell viability is reduced following invasion by PLD-expressing *A. haemolyticum*

**Figure 4:**
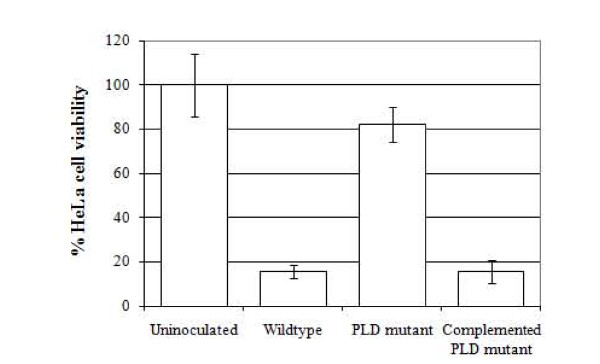
**PLD expressed inside HeLa cells is cytotoxic**. HeLa cells were inoculated with *A. haemolyticum *strains and the bacteria were allowed to adhere for 2 h and invade for 5 h prior to determination of host cell viability. Viability is shown as a percentage of that of diluent-treated cells, which was set to 100%. Error bars indicate one standard deviation from the mean calculated from the averages of at least three independent experiments conducted in triplicate.

The viability of HeLa cells following invasion by *A. haemolyticum *strains was measured to determine whether PLD expressed intracellularly was cytotoxic. The viability of *A. haemolyticum*-inoculated HeLa cells was determined as a percentage of uninoculated HeLa cells, which was set at 100%. Following invasion of host cells with wild type *A. haemolyticum*, only 15.6% of the HeLa cells remained viable after 5 h, compared with uninoculated HeLa cells (*p *< 0.05; Figure 4). The *pld *mutant displayed significantly reduced cytotoxicity with 82.3% of HeLa cells viable, as compared to the uninoculated control (*p *< 0.05; Figure 4). Invasion of HeLa cells with the complemented *pld *mutant resulted in 15.4% of HeLa cell viability, similar to that of the wild type (Figure 4). Initial measurements of HeLa viability at 2 h did not demonstrate a significant loss of host cell viability (data not shown). This is not unexpected, as time is required for the invaded bacteria to synthesize and express PLD, and for PLD to exert its effects leading to the end-stage, measurable outcome of loss of host cell viability.

These data indicated that invasion of HeLa cells by *A. haemolyticum *results in loss of host cell viability, with the majority of that being attributable to expression of PLD. Interestingly, when purified HIS-PLD was applied to the exterior of HeLa monolayers for 2-24 h, no HeLa cytotoxicity was detected over this time period, even at the highest concentrations of PLD (data not shown).

### *A. haemolyticum *PLD expressed inside HeLa cells results in host cell necrosis

**Figure 5:**
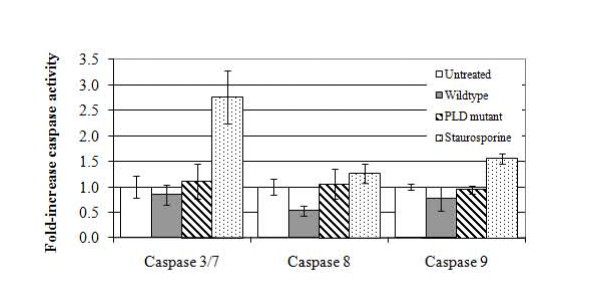
**Intracellular PLD does not initiate apoptosis in HeLa cells**. HeLa cells were inoculated with *A. haemolyticum *strains and the bacteria were allowed to adhere for 2 h and invade for 5 h prior to measurement of caspase 3/7, 8 or 9 activity. Activity is shown as a fold-change of untreated cells, which was set at a nominal value of 1.0. Error bars indicate one standard deviation from the mean calculated from the averages of at least three independent experiments conducted in triplicate.

The mechanisms of host cell death following invasion of wild type *A. haemolyticum *were investigated. Apoptosis was determined by measurement of caspases 3/7, 8 and 9 activity, following inoculation of HeLa cells with *A. haemolyticum *strains. The levels of caspase 3/7, 8 or 9 activation of untreated HeLa cells were set at a nominal value of 1.0-fold caspase activity (Figure 5), and the values of caspase activation in HeLa cells inoculated with *A. haemolyticum *strains were compared to this. Staurosporine (1 μM), used as a positive control, was able to induce apoptosis, as measured by 2.76-fold, 1.27-fold and 1.56-fold increases in caspase 3/7, 8 and 9 activities, respectively (*p *< 0.05; Figure 5). HeLa cells inoculated with wild type *A. haemolyticum *displayed no increase in apoptosis, as measured by caspase 3/7 or 9 activity (1.12-fold and 0.95-fold increases, respectively; Figure 5). However, HeLa cells inoculated with wild type *A. haemolyticum *had significantly reduced caspase 8 activity when compared to untreated cells (0.54-fold activity; *p *< 0.05; Figure 5). HeLa cells inoculated with the *pld *mutant also displayed similar levels of caspase 3/7, 8 and 9 expression as the uninoculated HeLa cells (0.85-fold, 1.06-fold and 0.77-fold, respectively; Figure 5). The caspase 3/7 assay was repeated at 1 or 24 h post-invasion, however, no significant differences were observed in activity of these caspases at these time points (data not shown). Therefore, it appears that invasion of HeLa cells with *A. haemolyticum *strains was unable to induce apoptosis under these conditions (Figure 5).

**Figure 6:**
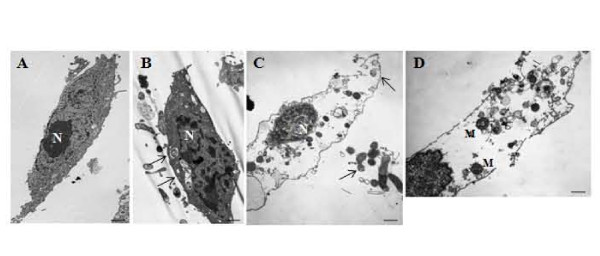
**PLD apparently induces host cell damage by necrosis**. Representative transmission electron micrographs of HeLa cells, (A) uninoculated, or inoculated with (B) *A. haemolyticum pld *mutant or (C, D) *A. haemolyticum *wild type using a standard invasion assay. Arrows indicate bacteria, N and M indicate the nucleus and mitochondria, respectively. The bars on the lower right indicates ~1 μm.

As bacterial invasion did not induce apoptosis, it suggested that loss of HeLa cell viability may be due to necrosis. HeLa cells were inoculated with *A. haemolyticum *strains and examined by TEM. Uninoculated, control HeLa cells displayed normal architecture (Figure 6A). HeLa cells inoculated with the *pld *mutant displayed typical cellular architecture; however, bacteria could be observed in membrane-bound vacuoles within some cells (Figure 6B). In contrast, wild type inoculated cells appeared necrotic, as there was no membrane integrity, the cytoplasm appeared to be absent, the nucleus was condensed and the mitochondria were swollen (Figure 6C, D), all of which are hallmarks of cellular necrosis. Bacteria could be observed both in proximity to, and inside, the HeLa cells, and intracellular bacteria were not found within vacuoles (Figure 6C).

## Discussion and Conclusions

Ceramides, including ceramide-1-PO_4_, are important mediators of a number of normal cellular signaling pathways such as cell growth, proliferation (including oncogenesis), apoptosis and inflammation via altered cytokine signaling [24]. While a number of bacteria express PLDs, there are only a few species expressing sphingomyelinases D, which specifically cleave SM to ceramide-1-PO_4 _in host cell membranes. Given the central role of PLDs in normal host cell physiology, it is easy to see how the dysregulated release of ceramides from SM by bacterial PLDs could potentially lead to pleomorphic effects on the host cell [24], and how these effects could benefit the infection process.

We report the first molecular characterization of the PLD (sphingomyelinase D) from *A. haemolyticum *and show that the action of this enzyme has implications in the pathogenesis of disease caused by this organism. In a manner analogous to host PLDs [38], *A. haemolyticum *PLD was able to stimulate reorganization of lipid rafts in epithelial cell plasma membranes in a dose-dependent manner (Figure 2C). This PLD-mediated lipid raft reorganization could be inhibited by anti-PLD antibodies, as well as by cholesterol sequestration (Figure 2D).

Recently, bacterially-induced lipid raft reorganization has been implicated in promoting efficient bacterial invasion rather than adhesion [39-42]. However, we observed that lipid raft rearrangement, mediated by PLD, directly promoted attachment to host cells, as an *A. haemolyticum pld *mutant had a 60.3% reduced adhesion as compared to the wild type (Figure 3A). It is unlikely that PLD, a secreted enzyme, acts directly as an adhesin. Furthermore, the hypothesis that PLD exposes a cryptic receptor, as seen with arcanobacterial neuraminidases [43], was also discarded as cholesterol sequestration by MβCD, which inhibits lipid raft rearrangement, also significantly reduces adhesion of *A. haemolyticum *to host cells (Figure 3A). A more likely explanation is that PLD-mediated lipid raft reorganization leads to protein clustering and increased local receptor concentrations [20], which in turn leads to enhanced bacterial adhesion. The nature of the host receptor and the adhesin on the bacterial cell is unknown, but the *A. haemolyticum *genome encodes at least one extracellular matrix binding (MSCRAMM) protein (B.H. Jost and S.J. Billington, unpublished data), which are known bacterial adhesins [44].

Expression of PLD by *A. haemolyticum *appears to negatively affect the ability of this organism to invade host cells, as the *pld *mutant has a more than 2-fold increased ability to invade HeLa cells as compared to the wild type (Figure 3B). We hypothesized that rather than directly affecting invasion, invasion of host cells with *A. haemolyticum *strains expressing PLD had detrimental effects, such as loss of host cell viability. Cell death would expose bacteria to the extracellular gentamicin, leading to an observed increase in invasion with the *pld *mutant.

This appeared to be the case, as PLD expressed from wild type *A. haemolyticum *inside host cells resulted in 84.4% loss of cell viability as compared to untreated cells (Figure 4). This is in contrast to host cells invaded by the *pld *mutant, which had only a 17.7% loss of viability (Figure 4). Interestingly, when recombinant PLD is applied to the exterior of the host cell, it did not cause cytotoxicity, as measured by cell viability. This is not surprising in that PLD alone is unable to cause sufficient membrane perturbations to lyse non-nucleated cells such as erythrocytes [45]. Proper bacterial delivery of PLD to the host cell seems to be required for effects on host cell viability.

Apoptosis was not detected following *A. haemolyticum *invasion of HeLa cells (Figure 5). Of all the organelles, the outer leaflet of the mitochondrial membrane is particularly rich in SM [17], and we hypothesized that PLD may target this structure, possibly leading to caspase 9 activation as part of the mitochondrial apoptosis pathway. However, caspase 9 activation was not detected following *A. haemolyticum *invasion of HeLa cells, nor was the activation of caspase 3/7 or 8, which are measures of general apoptosis or the extrinsic apoptosis pathway, respectively. We note that the findings from these apoptosis studies must be tempered with caution in that they were performed in a cell line, and may not accurately reflect what is occurring in host tissue.

The TEM study confirms the intracellular invasion of HeLa cells by *A. haemolyticum *and indicates that the *pld *mutant is unable to escape the invasion vacuole, at least by the measured time point (Figure 6B). In contrast, the wild type is able to escape the vacuole (Figure 6C) and can cause host cell death (Figure 4), apparently by necrosis (Figure 6C, D). Direct measurement of necrosis has been difficult, and has traditionally used changes to cellular architecture rather than specific bio-markers. However, better data is emerging about of the types of cell processes that initiate necrosis within the host cell, and recently it was determined that PLD-mediated release of ceramides can play a central role in initiating cellular necrosis [46].

Necrosis as a cause of host cell death may not be surprising given that a hallmark of *A. haemolyticum *pharyngitis is localized inflammation [2]. Necrosis-induced inflammation may enhance the immune response or cause localized tissue damage which promotes bacterial dissemination. The balance of these possibilities may be tipped towards bacterial invasion in the case of individuals who are also immunocompromised, elderly or have other co-morbid factors, leading to the more invasive sequelae observed with *A. haemolyticum *infections in this patient population [8-13].

From these studies we conclude that PLD expressed by *A. haemolyticum *is responsible for efficient host cell adhesion by reorganizing lipid rafts, which presumably clusters adhesin receptors. However, the identity of the host and bacterial receptors are currently unknown. PLD is also required following invasion into host cells. The *pld *mutant appears to be defective in that it cannot or is significantly delayed in its ability to escape the invasion vacuole, which leads to increased host cell viability. In contrast, the PLD-expressing wild type *A. haemolyticum *presumably escapes the vacuole, and PLD expressed inside the host cell causes cellular necrosis. The mechanism(s) by which *A. haemolyticum *PLD acts to cause necrosis are unknown. Host PLDs have a plethora of activities inside the cells [24], and dysregulated expression of bacterial PLD could lead to pleomorphic effects, any number of which could lead to the cellular signals for necrosis. Alternatively, PLD could trigger a specific necrotic response in the cell or PLD could actively block apoptosis, leading to a "forced" necrosis pathway [46]. Which of these hypotheses is correct remains to be elucidated with further study.

## Methods

### Bacterial strains and growth conditions

The type strain of *A. haemolyticum *(ATCC9345) was used for all experiments. The other *A. haemolyticum *strains were clinical isolates (n = 52) obtained from either throat or wound swabs and were grown on tryptic soy (TS) agar plates supplemented with 5% bovine blood at 37°C and 5% CO_2 _or in TS broth supplemented with 10% newborn calf serum (Atlas Biologicals) at 37°C with shaking. *Escherichia coli *DH5αMCR strains (Gibco-BRL) were grown on Luria-Bertani (LB) agar or in LB broth at 37°C. Antibiotics were added as appropriate: for *A. haemolyticum*, kanamycin (Kn) at 200 μg/ml, chloramphenicol (Cm) at 5 μg/ml; for *E. coli*, ampicillin at 100 μg/ml, Cm at 30 μg/ml, Kn at 50 μg/ml.

PLD production by *A. haemolyticum *isolates was identified by the presence of synergistic hemolysis following growth on TS agar plates with 5% bovine blood and 10% Equi Factor, as PLD is not hemolytic alone. Equi Factor was prepared from the 0.2 μm filtered supernatant of an overnight culture of *Rhodococcus equi *ATCC6939 [45]. Samples of *A. haemolyticum *ATCC9345 broth culture were harvested at points throughout the growth cycle. Culture supernatants were obtained by centrifugation and 0.2 μm filtration, and stored at -80°C prior to assay for PLD activity. Wells were punched into TS agar containing 5% bovine blood and 10% Equi Factor and 20 μl of culture supernatant was added. Zones of hemolysis were measured after 4 h incubation at 37°C.

### DNA techniques and sequence analysis

*E. coli *plasmid DNA extraction, DNA restriction, ligation, transformation, agarose gel electrophoresis and Southern transfer of DNA were performed as described [47]. Genomic DNA isolation and electroporation-mediated transformation of *A. haemolyticum *strains was performed as previously described for *A. pyogenes *[48], except that a capacitance of 25 μF and a resistance of 200 Ω were used. PCR DNA amplification was performed using *Taq *or Vent DNA polymerase (NEB) with the supplied reaction buffer for 35 cycles consisting of 1 min at 94°C, 1 min at 55°C, and 1 min/kb at 72°C. Preparation of DNA probes, DNA hybridization, and probe detection were performed using a DIG DNA Labeling and Detection Kit (Roche).

Database searches were performed using the BlastX and BlastP algorithms [49]. tRNA sequences were identified using the tRNAscan-SE program [50]. Signal sequence prediction was performed using SignalP [51]. Transcriptional terminators were identifier using mfold [52].

### Cloning and purification of a recombinant, 6xHis tagged-PLD (HIS-PLD)

The *pld *gene, lacking the signal sequence coding region, was amplified from *A. haemolyticum *ATCC9345 genomic DNA by PCR with a 5' primer containing a *Bam*HI site (5'-CGGCTGCGGATCCACTTGCGCAAGAACAACC-3') and a 3' primer containing an *Eco*RI site (5'-ATAAGAATTCGTGTTATCTCATTCG-3'; underlined in sequence). These primers amplified an 886-bp product from bases 94-940 of the *pld *gene, which was cloned into pTrcHis B (Invitrogen) to generate pBJ31, encoding HIS-PLD. Cultures for purification of HIS-PLD were grown to an OD_600 _= 0.6 prior to induction with 2.5 mM IPTG for 3 h and harvested by centrifugation. Cells were solubilized in 8M urea at 4°C overnight with gentle agitation. HIS-PLD was purified from the soluble material using TALON metal affinity resin (Clontech), and eluted from the resin with 150 mM imidazole in 20 mM Tris-HCl, 100 mM NaCl, pH 8.0. Purified HIS-PLD was mixed 1:1 with SDS-sample buffer and boiled for 5 min prior to electrophoresis in a 10% (w/v) SDS-polyacrylamide gel [47]. Proteins were transferred to nitrocellulose and Western blots were immunostained using rabbit anti-HIS-PLD (prepared by immunization of a rabbit with HIS-PLD; Antibodies Inc.) and goat anti-rabbit IgG(H+L)-peroxidase conjugate (KPL) as the primary and secondary antibodies, respectively [47]. SDS-PAGE and Coomassie Blue staining of purified HIS-PLD yielded a band of approximately 35.5-kDa and showed greater than >95% purity. Antiserum against PLD, but not pre-immune antiserum, reacted specifically with HIS-PLD (data not shown). Purified HIS-PLD retained hemolytic activity as demonstrated by PLD activity assay (data not shown). Total protein concentration was determined with Bradford protein assay reagent (Bio-Rad). Endotoxin contamination of HIS-PLD preparations was determined using the Limulus Amebocyte Lysate Pyrogent Kit (Cambrex), and endotoxin levels were negligible (<0.06 EU/ml; data not shown).

### Construction of a *pld *knockout mutant and a complementing plasmid

The *pld *gene was amplified from *A. haemolyticum *ATCC9345 by PCR using forward and reverse primers (5'-GTGTAAGCTTCAACATAGAGACATGG-3') and (5'-ATAAGAATTCGTGTTATCTCATTCG-3'). The PCR product was digested with *Hin*dIII-*Eco*RI using restriction sites engineered into the primers (underlined in sequence) and cloned into similarly digested pBC KS (Stratagene), to construct pBJ29. The *pld *gene in pBJ29 was interrupted by insertion with a 1.4-kb *Bam*HI fragment carrying the Kn resistance gene from pKRP11 [53], which allows for the construction of non-polar mutations. This plasmid was used to transform *A. haemolyticum *ATCC9345, selecting for Kn^R^Cm^S ^colonies. Southern blot analysis of *A. haemolyticum *wild type and *pld*- mutant genomic DNA confirmed inactivation of the *pld *gene via a double cross-over event (data not shown). A *pld *complementing plasmid, pBJ61, was constructed by cloning the insert of pBJ29 into pJGS180 [43], which replicates in *A. haemolyticum *(data not shown).

### Tissue culture cell adhesion and invasion assays

HeLa cells were cultured in Iscove's Modified Dulbecco's Medium with 10% fetal calf serum (IMDM-10% FCS) with 10 μg/ml gentamicin at 37°C and at 5% CO_2_. For adhesion assays, cells in IMDM-10% FCS, without gentamicin, were seeded into 24-well plates at 2 × 10^5 ^cells/well in 1 ml volumes. The cells were incubated overnight prior to the addition of log-phase *A. haemolyticum *at a multiplicity of infection (MOI) of 10:1. Bacterial adhesion was assessed after 2 h at 37°C. Cell monolayers were washed three times with 0.1M phosphate-buffered saline, pH 7.2 (PBS) to remove non-adherent bacteria. Cell monolayers were lysed using 1 ml ice-cold 0.1% Triton X-100 for 10 min, and viable bacteria were enumerated by dilution plating. To assess the inhibitory affect of the cholesterol sequestering agent methyl-beta-cyclodextrin (MβCD; Sigma) on adhesion, 5 mM MβCD was added to HeLa cells for 40 min prior to addition of bacteria, as described above, and maintained at 5 mM in the medium for the duration of the experiment. To assess the effect of exogenous PLD, 312 ng HIS-PLD was added to HeLa cells for 10 min prior to the addition of bacteria, as described above.

For invasion assays, bacteria were added at an MOI of 20:1, were allowed to adhere and invade for 2 h, at which time the cell monolayers were washed three times with Hank's Balanced Salt Solution, and IMDM-10% FCS containing 10 μg/ml gentamicin was added to the wells. The plates were incubated for an additional 2 h to allow invasion and killing of extracellular bacteria. The monolayers were washed and internalized bacteria were recovered and enumerated as above.

### Epithelial cell cytotoxicity

The cytotoxicity of HIS-PLD for epithelial cells was determined using the CellTiter 96^® ^Aqueous One Solution Cell Proliferation Assay (Promega). HeLa cells were seeded into 96-well plates at 2 × 10^4 ^cells/well and the cells were incubated for 18 h to achieve 80% confluence. Triplicate wells were incubated with doubling dilutions of HIS-PLD (0-2 μg) and incubated for 2-24 h, as above. Dilutions of imidazole-containing HIS-protein elution buffer were used as a control. Additional monolayers were inoculated with log-phase *A. haemolyticum *strains at an MOI of 20:1, and incubated for 2 h, as above. The monolayers were washed three times with PBS and IMDM-10% FCS containing 10 μg/ml gentamicin was added and the cells were incubated for a further 5 h. 3-(4,5-dimethylthiazol-2-yl)-5-(3-carboxymethoxyphenyl)-2-(4-sulfophenyl)-2H-tetrazolium/phenazine methosulfate (MTS/PMS) reagent was added and the HeLa cells were incubated for an additional 3 h. The color change was measured at 492 nm using a Synergy HT plate reader (Bio-Tek). Determination of % cell viability was performed using the appropriate control values, as described by the manufacturer.

### Lipid raft labeling

HeLa cells were seeded into 8-well chamber slides (Lab-Tek) at 1 × 10^4 ^cells/well and were incubated overnight to achieve 70% confluence. The cells were washed with PBS prior to incubation with dilutions of HIS-PLD (0-50 ng) for 10 min at 37°C and 5% CO_2_. Dilutions of imidazole-containing elution buffer were used as a control. Lipid rafts were labeled using the Vybrant^® ^Lipid Raft Labeling Kit (Molecular Probes). The slides were mounted in 2% 1,4-diazabicyclo [2.2.2] octane (DABCO; Sigma) in 50% glycerol and visualized with a Nikon epifluorescence microscope fitted with a rhodamine filter.

To assess the inhibitory effect of specific antibody, 1/1000 dilutions of anti-PLD or pre-immune serum were incubated with 50 ng HIS-PLD for 1 h at 37°C prior to addition of the mixture to the HeLa cell monolayer. To assess the effect of cholesterol sequestration, 5 mM MβCD was added to HeLa cells for 40 min at 37°C and 5% CO_2 _prior to stimulation of the cells with 50 ng HIS-PLD. PLD enzymatic activity was not inhibited by the presence of 5 mM MβCD (data not shown).

### Transmission electron microscopy (TEM)

HeLa cell monolayers were inoculated and incubated as for the invasion assay described above. The cells were harvested by scraping and were fixed with 4% formaldehyde-1% glutaraldehyde in PBS, embedded in Epon-Araldite, postfixed with 1% osmium tetroxide and stained with 5% uranyl acetate. Thin sections (50 nm) were examined using a Philips CM-12 electron microscope at an accelerating voltage of 60 kV.

### Apoptosis assays

HeLa cells were seeded into 96-well plates at 2 × 10^4 ^cells/well and the cells were incubated overnight to achieve 80% confluence. Triplicate wells were inoculated with *A. haemolyticum *strains, as described above for the epithelial cell cytotoxicity assay. Apoptosis was measured using the Caspase-Glo 3/7, 8 or 9 Assay Systems (Promega). HeLa cells were incubated with 1 μM staurosporine (Sigma) to induce apoptosis, as a positive control.

### Statistical analysis

Statistical significance was determined at the *p *< 0.05 level with single factor ANOVA, calculated using Microsoft Excel.

### Nucleotide sequence accession number

The *pld *gene region sequence data were submitted to the GenBank/EMBL/DDBJ databases under accession number FJ766092.

## Authors' contributions

EL conducted the bulk of the experiments and wrote the first draft of the manuscript; SJB constructed the *pld *mutant and provided scientific discussion; PC provided clinical isolates. DJM edited and submitted the manuscript; BHJ did the initial characterization of PLD activity on RBCs, provided scientific guidance and discussion and wrote the completed manuscript. All authors read and approved the final manuscript.
